# *Sorbaria kirilowii* Ethanol Extract Exerts Anti-Inflammatory Effects In Vitro and In Vivo by Targeting Src/Nuclear Factor (NF)-κB

**DOI:** 10.3390/biom10050741

**Published:** 2020-05-10

**Authors:** Jiwon Jang, Jong Sub Lee, Young-Jin Jang, Eui Su Choung, Wan Yi Li, Sang Woo Lee, Eunji Kim, Jong-Hoon Kim, Jae Youl Cho

**Affiliations:** 1 Department of Integrative Biotechnology, Sungkyunkwan University, Suwon 16419, Korea; rhea980327@gmail.com; 2 Research Institute of Biomolecule Control and Biomedical Institute for Convergence at SKKU (BICS), Sungkyunkwan University, Suwon 16419, Korea; 3 DanjoungBio Co., Ltd., Wonju 26303, Korea; js.lee@danjoungbio.com (J.S.L.); esavella@hanmail.net (E.S.C.); 4 College of Veterinary Medicine, Chonbuk National University, Iksan 54596, Korea; jyj3010@daum.net; 5 Institute of Medicinal Plants, Yunnan Academy of Agricultural Sciences, Kunming 650224, China; wyli2012@126.com; 6 International Biological Material Research Center, Korea Research Institute of Bioscience and Biotechnology, Daejeon 34141, Korea; ethnolee@kribb.re.kr

**Keywords:** *Sorbaria kirilowii*, anti-inflammatory effect, NF-κB signaling pathway, IKKα/β Src

## Abstract

Inflammation is a fundamental process for defending against foreign antigens that involves various transcriptional regulatory processes as well as molecular signaling pathways. Despite its protective roles in the human body, the activation of inflammation may also convey various diseases including autoimmune disease and cancer. *Sorbaria kirilowii* is a plant originating from Asia, with no anti-inflammatory activity reported. In this paper, we discovered an anti-inflammatory effect of *S. kirilowii* ethanol extract (Sk-EE) both in vivo and in vitro. In vitro effects of Sk-EE were determined with lipopolysaccharide (LPS)-stimulated RAW264.7 cells, while ex vivo analysis was performed using peritoneal macrophages of thioglycollate (TG)-induced mice. Sk-EE significantly reduced the nitric oxide (NO) production of induced macrophages and inhibited the expression of inflammation-related cytokines and the activation of transcription factors. Moreover, treatment with Sk-EE also decreased the activation of proteins involved in nuclear factor (NF)-κB signaling cascade; among them, Src was a prime target of Sk-EE. For in vivo assessment of the anti-inflammatory effect of Sk-EE, HCl/EtOH was given by the oral route to mice for gastritis induction. Sk-EE injection dose-dependently reduced the inflammatory lesion area of the stomach in gastritis-induced mice. Taking these results together, Sk-EE exerts its anti-inflammatory activity by regulating intracellular NF-κB signaling pathways and also shows an authentic effect on reducing gastric inflammation.

## 1. Introduction

Inflammation is an innate immune process of eliminating foreign pathogens and repairing damaged body tissues or cells. The overall process of inflammation is generally carried out by two types of immune cells: adaptive cells such as B or T lymphocytes, and innate immune cells including dendritic cells and macrophages [1,2,3]. Among different triggers, microbial infection is one of the most common causes for the activation of the inflammatory process. Microbial species possess conserved structures on their cell membrane or cell wall, called pathogen-associated molecular patterns (PAMPs), which are recognized by inflammation-related receptors. Pattern recognition receptors (PRRs), are one kind of these receptors, which are responsible for the detection of PAMPs and the initiation of intracellular signaling process for the onset of inflammation [4,5,6]. Toll-like receptors (TLRs) are transmembrane proteins that include one demonstrative family of PRRs; in humans, 10 different TLRs have been identified. Lipopolysaccharide (LPS), a type of PAMPs from Gram-negative bacteria, is a potential ligand for TLR4. However, some TLRs—including TLR4—require additional proteins for the recognition of LPS. CD14, a GPI-anchored protein, aids binding of LPS to TLR4 along with LPS-binding protein (LBP) [7]. The recognition of LPS by TLR4 initiates sequential intracellular signaling processes critical for inflammation [4,8,9,10,11].

Cellular responses to external or internal stimuli are predominantly regulated by intracellular signaling processes [12]. Likewise, inflammation also activates several intracellular signaling pathways [13]. Among them, the nuclear factor-kappa B (NF-κB) pathway is one of the most critical pathways activated by inflammatory stimuli, resulting in a variety of inflammatory responses [14,15]. Initiated from the membrane receptor TLR4, a series of protein kinases are involved in the signal transduction. These proteins are activated via phosphorylation by their upstream kinases and, in turn, activate their downstream molecules by phosphorylation [16,17,18]. The NF-κB signaling pathway is composed of tyrosine protein kinases Src/Syk, IκB kinase α/β (IKK α/β, and NF-κB inhibitor, α (IκBα). Activated Src or Syk family phosphorylates phosphoinositide-3-kinase (PI3K) and IKK α/β, which, in turn, phosphorylates IκBα [19]. In unstimulated cells, NF-κB is bound with its inhibitor IκBα in the cytoplasm. Once stimulated, phosphorylated IκBα is then ubiquitinated by ubiquitin ligases and degraded by proteasomes; finally, NF-κB subunits are released and translocated into the nucleus [13,14,15,18,19,20,21]. Activated NF-κB allows for the transcription of proinflammatory cytokines such as interleukin 1β IL-1β) and enzymes such as cyclooxygenase-2 (COX-2) and inducible nitric oxide (NO) synthase (iNOS) to produce inflammatory mediators including prostaglandin E_2_ (PGE_2_) and NO, respectively. These factors together promote the inflammatory process and induce other immune cells to function [22,23,24,25]. Despite the importance of the immune response, failed resolution of inflammation may increase the risk of several diseases, including cancer and autoimmune diseases. Therefore, proper regulation of inflammation is critical for maintaining physiological homeostasis, and the development of effective anti-inflammatory drugs is hence strongly required [26].

*Sorbaria kirilowii* (Regel) Maxim. (Sk-EE), a species of the Rosaceae family, is a flowering plant distributed in temperate areas of Asia, including China and Korea [27]. Several reports have revealed that the *Sorbaria* genus may possess antioxidative activity and may also prevent cancer proliferation and chronic liver damage [28,29,30]. Nevertheless, there is no research available concerning its inflammation-regulatory activity. Therefore, in this study, we investigated the novel anti-inflammatory effect of Sk-EE both in vitro and in vivo, focusing on the immunoregulating pathways and molecular mechanisms.

## 2. Materials and Methods

### 2.1. Materials

First, 95% ethanol extract of Sk-EE was obtained from the Korea Plant Extract Bank (Cheongju, Korea). Briefly, dried and refined leaves and twigs of *Sorbaria kirilowii* (100 g) were extracted with 1 L of 95% ethanol for 2 h, twice. The extract was percolated with filter paper (3 mm; Whatman PLC, Kent, UK), condensed using a Buchi rotary evaporator (Merck, Darmstadt, Germany) and lypophilized using a laboratory freeze dryer (Martin Christ Gefriertrocknungsanlagen GmbH, Harz, Germany). *N*(G)-Nitro-l-arginine methyl ester (l-NAME), ranitidine, LPS, silibinin, genistein, quercetin, kaempherol, polyethylene imidazole (PEI), (3-4,5-dimethylthiazol-2-yl)-2,5-diphenyltetrazolium bromide (MTT), and sodium dodecyl sulfate (SDS) were purchased from Sigma Chemical Co. (St. Louis, MO, USA). Dulbecco’s Modified Eagle’s medium (DMEM) and Roswell Park Memorial Institute (RPMI) 1640, Fetal bovine serum (FBS), phosphate-buffered saline (PBS), and TRIzol reagent were purchased from GIBCO (Grand Island, NY, USA). RAW264.7 cells (no. TIB-71) and HEK293 cells (no. CRL-1573) were purchased from the American Type Culture Collection (Rockville, MD, USA). Antibodies specific for phosphorylated or total forms of p65, p50, LaminA/C, p85, IKK α/β, IκBα, Syk, Src, β-actin, HA, Myc, rabbit IgG, and Mouse IgG were purchased from Cell Signaling Technology (Danvers, MA, USA). The host, concentrations, and exposure times of antibodies are listed in Table 1.

### 2.2. Animals, Cell Culture, and Compound Preparation

Institute of Cancer Research (ICR) mice (male, 6–8 weeks old) were purchased from Daehan Biolink (Osong, Korea) and housed in plastic cages under standard conditions. Water and feed (Samyang, Daejeon, Korea) were given ad libitum. All studies were conducted according to the guidelines of the Institutional Animal Care and Use Committee at Sungkyunkwan University (Suwon, Korea; approval ID: 2019-12-03-1).

Murine macrophage cell line RAW264.7 cells and human kidney cell line HEK293T cells were cultured in RPMI1640 and DMEM culture media containing streptomycin (100 µg/mL), penicillin (100 IU/mL), and 5% or 10% inactivated FBS. Cells were cultured in a humidified incubator, which maintains an environment of 5% CO_2_ with a temperature of 37 °C. A stock solution of Sk-EE (100 mg/mL) was prepared using DMSO. The stock solution was further diluted to 50 to 200 µg/mL using cell culture media for in vitro and ex vivo studies. For in vivo studies, the stock solution of Sk-EE was diluted to 100 to 200 mg/kg using a 0.5% sodium carboxymethyl cellulose (CMC) solution [31,32].

### 2.3. Peritoneal Macrophages Preparation

Five ICR mice (male, six weeks old) underwent four days of refinement. Inflammation was then induced by intraperitoneal injection of 4% sterile thioglycolate (TG) broth. After four days of induction, the mice were sacrificed, and peritoneal macrophages were harvested using PBS. Exudates were washed with red blood cell (RBC) lysis buffer for complete RBC lysis [33]. Obtained peritoneal macrophages were maintained in RPMI 1640 media containing streptomycin (100 µg/mL), penicillin (100 IU/mL), and 10% inactivated FBS and were cultured in the humidified incubator with 5% CO_2_ environment and temperature of 37 °C.

### 2.4. NO Production Assay

RAW264.7 cells (1 × 10^5^cells/mL) and peritoneal macrophages (3 × 10^5^ cells/mL) were seeded in 96-well culture plates and incubated for 24 h. Sk-EE (50–200 µg/mL) or standard compound l-NAME (0.5–1 mM) were pretreated 30 min before inflammatory induction. LPS (1 µg/mL) was applied to induction groups and cells were incubated for 24 h. The NO production of the cells was assessed with Griess reagent and the optical density (OD) was measured at 540 nm. Optical densities of each group were calculated to NO concentration according to the standard curve, then were analyzed as percent of control (normal group) [34].

### 2.5. Cell Viability Assay

RAW264.7 cells (1 × 10^5^ cells/mL), peritoneal macrophages (3 × 10^5^ cells/mL) and HEK293T cells (1 × 10^5^ cells/mL) were seeded in 96-well culture plates and incubated for 24 h. Sk-EE (50–200 µg/mL) or standard compound l-NAME (0.5–1 mM) was given and the cells were incubated for 24 h. The MTT solution was applied to cells and, after three hours, the MTT stopping solution was dispensed. After 24 h of incubation, cell viability was assessed at 570-nm OD [35].

### 2.6. High-Performance Liquid Chromatography (HPLC) Analysis

HPLC analysis was utilized for the identification of phytochemical characteristics of Sk-EE. Silibinin, genistein, quercetin, and kaempferol were used as standard compounds, as previously reported [36,37]. Detailed conditions of HPLC analysis are shown in Table 2.

### 2.7. mRNA Expression Analysis by Reverse-Transcription Polymerase Chain Reaction (RT-PCR)

RAW264.7 cells (1 × 10^6^ cells/mL) were seeded in 12-well culture plates and incubated for 24 h. Cells were pretreated with Sk-EE (100 and 200 µg/mL) before LPS (1 µg/mL) application. After six hours of LPS induction, cells were harvested using cold PBS and total cell RNA was extracted using Trizol reagent. The quantification and analysis of mRNA expression levels of iNOS, COX-2, and IL-1β were evaluated by semiquantitative RT-PCR and agarose gel electrophoresis, respectively. Relative intensities of PCR bands were analyzed using ImageJ program [38]. The sequences of primers used in this study are listed in Table 3.

### 2.8. Plasmid Transfection and Luciferase Reporter Assay

HEK293T cells (1.2 × 10^5^ cells/mL) were seeded in 24-well culture plates and incubated for 24 h. Target gene constructs (i.e., Tag2-MyD88, Tag2, CFP-TRIF, CFP, NF-κB-luc, and β-gal) were transfected into cells using PEI solution. After 24 h of incubation, cells were treated with luciferase lysis buffer and kept at −70 °C for three hours. The expression of NF-κB-luc was assessed using luminescence and β-gal was measured at 405 nm [39].

### 2.9. Total and Nuclear Cell Lysate Preparation

RAW264.7 cells (2.5 × 10^6^ cells/mL or 75% confluency) were seeded in 3-cm or 10-cm culture plates and incubated for 24 h. Cells were pretreated with Sk-EE (200 µg/mL) before LPS (1 µg/mL) stimulation. At certain time points, cells were harvested using cold PBS. Total cell lysates were prepared by lysing the cells with cold lysis buffer (20 mM of Tris-HCl, pH: 7.4; including 2 mM of EDTA, 2mM of EGTA, 50 of mM glycerol phosphate, 1 mM of DTT, 2 µg/mL of aprotinin, 2 µg/mL of leupeptin, 1 µg/mL of pepstatin, 50 µM of PMSF, 1mM of benzamide, 2% Triton X-100, 10% glycerol, 0.1 mM of sodium vanadate, 1.6 mM of pervanadate, and 20 mM of NaF) and centrifugated at 12,000 rpm for five minutes. Nuclear lysates were prepared by treating with homogenization buffer A (20 mM of Tris-HCl pH: 8.0; including 10 mM of EGTA, 2 mM of EDTA, 2 mM of DTT, 1 mM of PMSF, 25 µg/mL of aprotinin, and 10 µg/mL of leupeptin) followed by sonication, which was used to lyse cells. After centrifugation at 8000 rpm for 15 min, the supernatant (cytosolic fraction) was transferred, while the remaining pellet (nuclear fraction) was treated with homogenization buffer B (1% Triton X-100 in homogenization buffer A and vortexed for complete lysis. Total and nuclear lysates were kept at −20 °C until use [37,40,41].

### 2.10. Western Blot Analysis

The protein concentration of total or nuclear lysates of cells was measured at OD 570 nm and sampled into quantified amounts for each group. Samples were size-dependently separated through SDS-polyacrylamide gel electrophoresis (30% acrylamide, Tris-base, 10% SDS, 10% APS, TEMED; pH: 8.8) in SDS-PAGE running buffer (10% SDS, Tris-base, glycine). Separated proteins were transferred onto PVDF membranes in transfer buffer (10% SDS, Tris-base, glycine, methanol). Blocking of membranes was performed at room temperature for one hour, with 5% BSA solution. Primary antibodies specific to target proteins (total or phosphorylated p65, p50, IKK α/β, IκBα, Syk, Src, p85, β-actin, p65, p50, and Lamin A/C) were incubated with the membrane in 5% BSA solution at room temperature for one to two hours, or overnight at 4 °C. After washing three times with 0.1% TBST (Tris-base, NaCl, 0.1% Tween 20; pH: 7.6), HRP-attached secondary antibodies specific to the primary antibodies were incubated with the membrane in 5% BSA solution at room temperature for one to two hours, or overnight at 4 °C. Protein visualization was achieved by detecting chemiluminescence signal produced by ECL reagent, with ChemiDoc XRS (Bio-Rad, Hercules, CA, USA.) Relative intensities of protein bands were analyzed using ImageJ program. [37,39].

### 2.11. Cellular Thermal-Shift Assay

HEK293T cells (3 × 10^5^ cells/mL) were seeded in six-well culture plates and incubated for 24 h. Genes (Myc-*Syk* and HA-*Src*) were then transfected to the cells using PEI. After 24 h of incubation, Sk-EE (200 µg/mL) was treated with new media and cells were incubated for an additional 24 h. Cells were harvested using trypsin and centrifuged at 1000 rpm for five minutes. Pellets were washed with PBS three times and cell counting was conducted for quantification. At this point, 1.5 × 10^6^ cells were sampled for each group and a temperature shift was achieved using real-time PCR (43–64 °C, three minutes for each temperature). After freezing and thawing with liquid nitrogen for three times and centrifugation, 5× sample buffer was added to supernatant of each sample. The analysis of protein expression level was completed through Western blotting and detection using ECL reagents [42].

### 2.12. HCl/EtOH-Induced Acute Gastritis in ICR Mice

ICR mice (male, six weeks old) were subjected to four days of refinement and one day of starvation before undergoing compound injection. The weights of these mice were measured for proper drug administration. Sk-EE (100 and 200 mg/kg, according to in vitro target concentration) and the control compound ranitidine (40 mg/kg) were orally administered three times a day for two days. Acute gastric inflammation was induced by the oral injection of HCl/EtOH. After one hour of induction, the mice were sacrificed, and their stomachs were extracted and washed with PBS three times. Inflammatory lesions were photographed with a white background. Stomach samples were ground in liquid nitrogen and lysed with cold lysis buffer (20 mM Tris-HCl, pH: 7.4; including 2 mM of EDTA, 2 mM of EGTA, 50 mM of glycerol phosphate, 1 mM of DTT, 2 µg/mL of aprotinin, 2 µg/mL of leupeptin, 1 µg/mL of pepstatin, 50 µM of PMSF, 1 mM of benzamide, 2% Triton X-100, 10% glycerol, 0.1 mM of sodium vanadate, 1.6 mM of pervanadate, and 20 mM of NaF). Protein expression levels of the samples were assessed by Western blotting and detection was completed using ECL reagents [43].

### 2.13. Statistical Analysis

All experimental data in this paper are presented as mean ± standard deviation of 3–6 replicates for each experiment. All results were analyzed by Mann–Whitney *U* tests for statistical comparisons. *p* values < 0.05 or 0.01 were considered statistically significant.

## 3. Results

### 3.1. Anti-Inflammatory Effect of Sk-EE In Vitro and Ex Vivo

**Figure 1:**
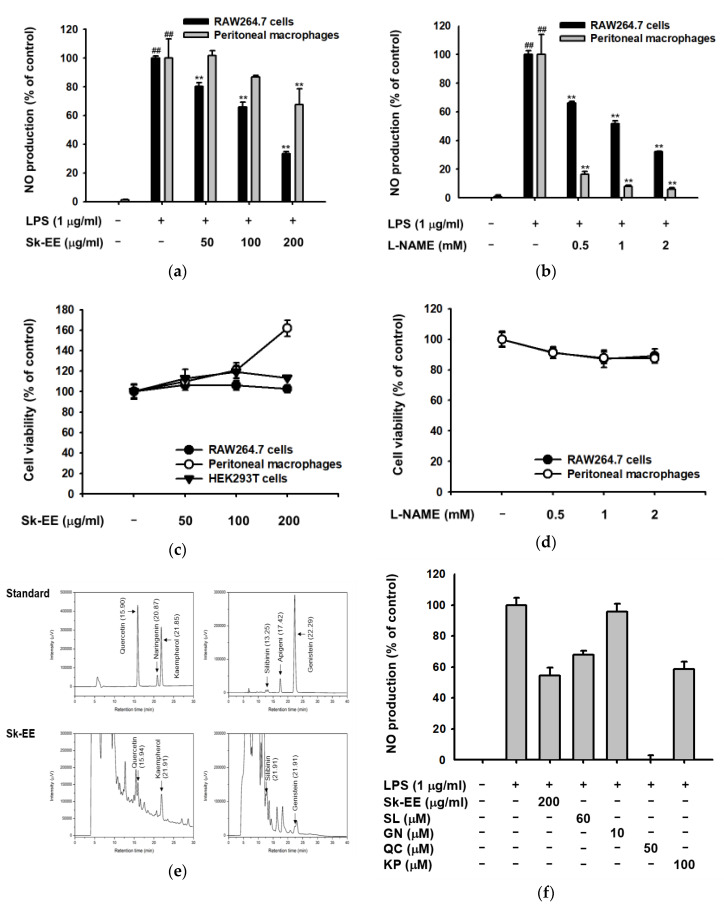
Effects of Sk-EE on nitric oxide (NO) production and its cytotoxicity analysis in macrophages. (**a**,**b**) RAW264.7 cells or peritoneal macrophages were pretreated with indicated doses of Sk-EE or l-NAME and induced by LPS (1 µg/mL) for 24 h. NO production was measured by Griess assay. (**c**,**d**) RAW264.7 cells, peritoneal macrophages, or HEK293T cells were treated with indicated doses of Sk-EE or L-NAME and their cell viability was determined by MTT assay. (**e**) Phytochemical characteristics of Sk-EE were analyzed via HPLC. (**f**) Detected flavonoids and Sk-EE were pretreated to RAW264.7 cells 30 min before LPS induction, and NO production levels were measured through Griess assay. # *p* < 0.05 and ## *p* < 0.01 compared to normal group; * *p* < 0.05 and ** *p* < 0.01 compared to control group. All data presented (**a**–**d**) are expressed as mean ± SD of experiments performed with 4 samples. SL: silibinin. GN: genistein. QC: quercetin. KP: kaempherol. +: treatment, −: no treatment.

To examine the anti-inflammatory effect of Sk-EE, NO production—which is one of the most common consequences of the inflammatory process—was measured from macrophages. LPS was used as the stimulating ligand for TLR4 in murine macrophage cell line RAW264.7 cells and mouse-derived peritoneal macrophages. Under LPS-induced conditions, NO production levels of RAW264.7 cells and peritoneal macrophages were dose-dependently reduced by indicated concentrations of Sk-EE treatment (Figure 1a). l-NAME, a nitric oxide synthesis inhibitor [44], was used as a positive control. l-NAME (0.5, 1, and 2 mM) treatment also significantly reduced NO production in both RAW264.7 cells and peritoneal macrophages in a dose-dependent manner (Figure 1b), as previously reported. Moreover, to determine whether or not Sk-EE has a cytotoxic effect on cells, the cell viabilities of RAW264.7 cells, peritoneal macrophages, and HEK293T cells were measured. Ultimately, the viability of RAW264.7 cells and peritoneal macrophages remained over normal levels under indicated doses of Sk-EE treatment (Figure 1c). The control compound l-NAME did not cause cell death in RAW264.7 cells or peritoneal macrophages (Figure 1d). In addition, HPLC analysis of Sk-EE showed that Sk-EE includes silibinin, genistein, quercetin, and kaempferol, types of flavonoids which are known to have anti-inflammatory activity (Figure 1e). NO production levels of above flavonoids were also assessed with RAW264.7 cells for further demonstration of the inhibitory effect of Sk-EE on NO production. As shown in the result, most of the flavonoids detected by HPLC were able to decrease NO production (Figure 1f).

### 3.2. Anti-Inflammatory Effect of Sk-EE at the Transcriptional Level

**Figure 2:**
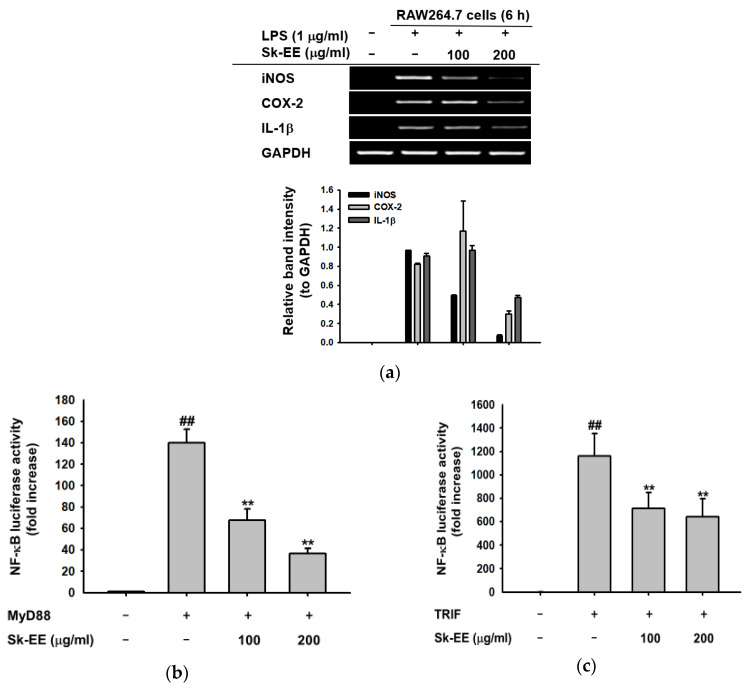

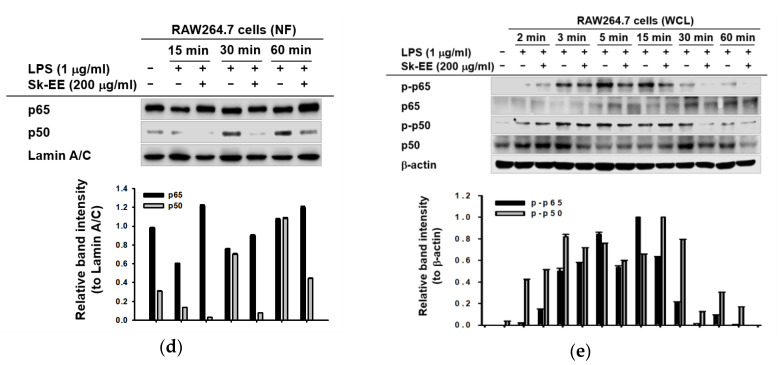
Effects of Sk-EE on the mRNA expression of inflammatory genes and nuclear translocation level of inflammatory transcription factors. (**a**) RAW264.7 cells were pretreated with indicated concentrations of Sk-EE and induced by lipopolysaccharide (LPS) (1 µg/mL) for six hours. mRNA expression levels of iNOS, COX-2, IL-1β, and GAPDH (as a control) were measured using reverse-transcription polymerase chain reaction (RT-PCR) and agarose gel electrophoresis. (**b**,**c**) HEK293T cells were transfected with NF-κB-luc, MyD88 or TRIF, and β-gal (as a control) genes by PEI transfection for 24 h, then treated with indicated doses of Sk-EE for 24 h. The expression of NF-κB was determined by luciferase assay. (**d**,**e**) RAW264.7 cells were pretreated with Sk-EE (200 µg/mL) for the indicated times and nuclear fractions or whole-cell lysates for detecting p65, p50, p-p50, p-p65, and Lamin A/C were analyzed by Western blotting. # *p* < 0.05 and ## *p* < 0.01 compared to normal group; * *p* < 0.05 and ** *p* < 0.01 compared to control group. Data presented (**b**,**c**) are expressed as mean ± SD of experiments performed with 6 samples. NF: nuclear faction. +: treatment, −: no treatment.

For the determination of an inhibitory effect of Sk-EE on inflammatory gene expression in macrophages, mRNA expression levels of proinflammatory cytokines were measured by reverse-transcription PCR using total cell RNA. Under LPS-treated conditions, Sk-EE (100 and 200 µg/mL) considerably reduced the mRNA expression of iNOS, COX-2, and IL-1β—three representative proinflammatory cytokines—in RAW264.7 cells (Figure 2a). Furthermore, in order to investigate the suppressive effect of Sk-EE on the inflammatory transcription factor NF-κB, we measured the activation level of the NF-κB promoter by monitoring its luciferase activity in HEK293T cells induced by MyD88 and TRIF, two adaptor molecules attached to TLR4. As shown in Figure 2, Sk-EE considerably repressed the activation of NF-κB in HEK293T cells induced by MyD88 (Figure 2b) and TRIF (Figure 2c). To facilitate a detailed examination, the nuclear translocation patterns of p65 and p50, which are two subunits composing NF-κB, were analyzed by western blotting using nuclear lysates of RAW264.7 cells. A lower protein level of p50 was detected in the Sk-EE–treated (200 µg/mL) group after 30 and 60 min of LPS induction in RAW264.7 cells (Figure 2d). To confirm this result, we also checked the level of phospho (p)-p50 and p-p65 from whole-cell lysates with their specific antibodies. Expectedly, phosphorylated p65 and p50 expressions were clearly reduced at 30 and 60 min after induction with LPS (Figure 2e). Therefore, we focused further on the anti-inflammatory effect of Sk-EE on the NF-κB signaling pathway.

### 3.3. Anti-Inflammatory Effect of Sk-EE on the NF-κB Signaling Pathway

**Figure 3:**
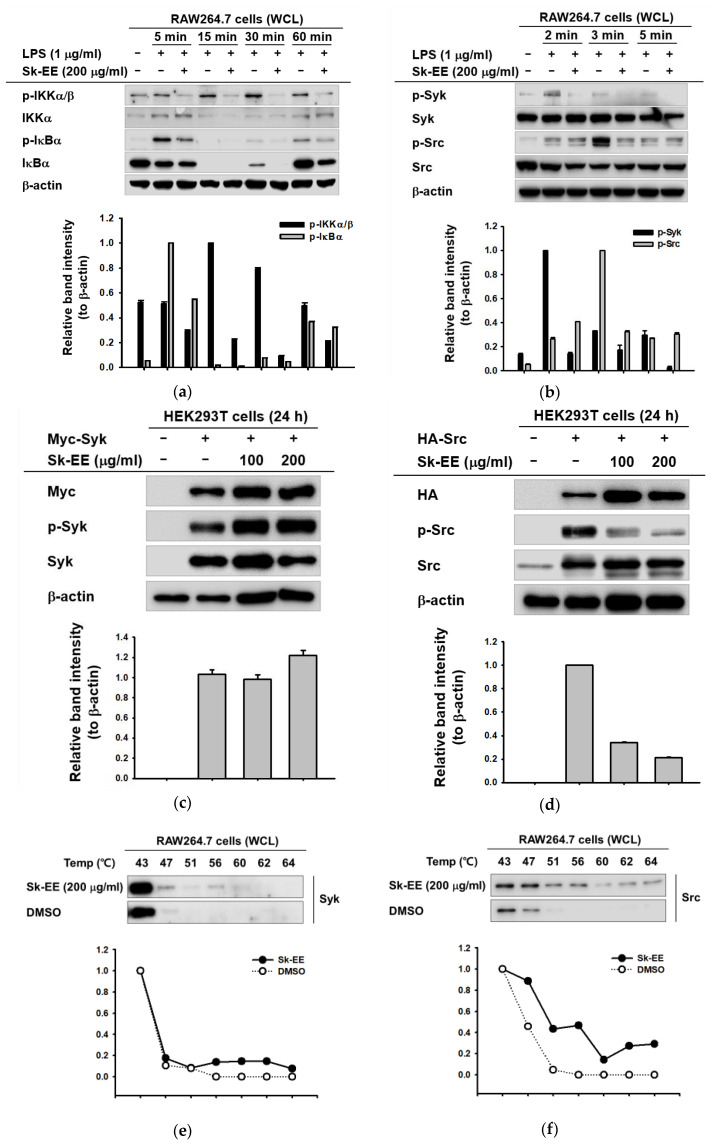
Effects of Sk-EE on the protein expression of the NF-κB signaling pathway and target proteins. (**a**,**b**) RAW264.7 cells were pretreated with Sk-EE (200 µg/mL) and induced by LPS (1 µg/mL) for the indicated time. The expression levels of total or phosphorylated forms of IKKα/β, IκBα, Syk, Src, and control protein β-actin were analyzed through western blotting. (**c**,**d**) HEK293T cells were transfected with Myc-Syc or HA-Src by PEI transfection for 24 h and treated with indicated doses of Sk-EE for 24 h. The protein expression levels of Myc, Syk, p-Syk, HA, Src, p-Src, and the control protein β-actin were analyzed by western blotting. (**e**,**f**) RAW264.7 cells were transfected with Myc-Syk or HA-Src through PEI transfection for 24 h and treated with Sk-EE (200 µg/mL) for 24 h. Binding affinities of Sk-EE and Syk or Src were determined by cellular thermal shift assay. WCL: whole-cell lysate. +: treatment, −: no treatment.

To verify the repressive effect of Sk-EE on upstream molecules of NF-κB, we examined the phosphorylation levels of the proteins involved in the NF-κB signaling pathway by western blotting, using whole lysates of LPS-stimulated RAW264.7 cells. First of all, since NF-κB constituents showed reducing pattern in nuclear fraction of cells when inflammation was induced (Figure 2d), we further assessed their expression patterns in whole-cell level. Therefore, we were able to investigate their upstream kinases; phosphorylated and total forms of IκBα and IKK α/β, two upstream regulators of NF-κB, were analyzed. The results showed that the phosphorylation of IκBα was clearly reduced by Sk-EE (200 µg/mL) treatment after five to 60 min of LPS induction; meanwhile, IKKα/β also showed inhibited patterns of its phosphorylated forms (Figure 3b). Consequently, we further examined the effects of Sk-EE on Syk and Src, two upstream molecules of IκBα and IKKα/β. Under LPS-treated conditions, Sk-EE (200 µg/mL) evidently inhibited phosphorylated Syk at two to five minutes after induction, whereas phosphorylated Src displayed a reducing pattern only at the three-minute time point (Figure 3c). To determine the prime target of Sk-EE, we analyzed the phosphorylation levels of Syk and Src under Syk- or Src-overexpressed conditions in HEK293T cells by cellular thermal-shift assay. While the expression of phosphorylated Syk did not show a reducing pattern after Sk-EE (100 or 200 µg/mL) treatment (Figure 3d), the phosphorylated Src level was decreased by Sk-EE in a dose-dependent manner (Figure 3e). Correspondingly, the assessment of binding of Sk-EE (200 µg/mL) on Syk revealed that there is low interaction between the two molecules (Figure 3f), whereas Sk-EE achieved strong binding to Src protein to retain stability (Figure 3g). These data together suggest that Sk-EE regulates two proteins in inflammatory responses, i.e., the Src and Syk kinases, by direct or indirect ways, respectively.

### 3.4. Anti-Inflammatory Effect of Sk-EE In Vivo

**Figure 4:**
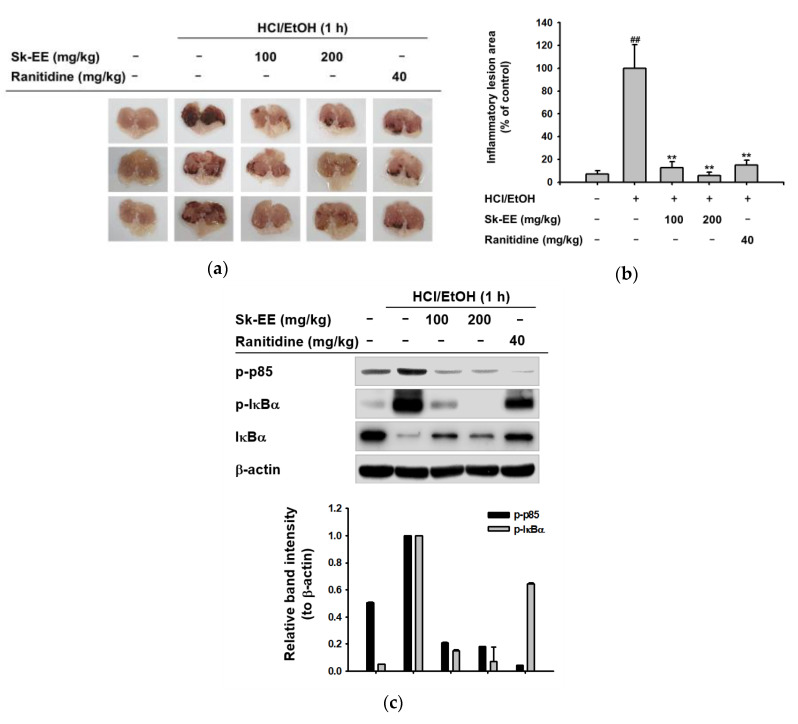
In vivo effects of Sk-EE on HCl/EtOH-induced gastritis mice. (**a**) The indicated doses of Sk-EE and control compound ranitidine were orally injected into mice three times in two days. HCl/EtOH was orally administered to mice one hour before sacrifice. The stomachs of sacrificed mice were isolated and inflammatory lesions were photographed. (**b**) Inflammatory lesion areas of each group were numerically analyzed by ImageJ (National Institutes of Health, Bethesda, MD, USA) and graphed. (**c**) The stomach samples of the gastritis mouse model were ground and lysed with buffer. Protein levels of total and phosphorylated forms of IκBα and control protein β-actin were analyzed by western blotting. # *p* < 0.05 and ## *p* < 0.01 compared to normal group; * *p* < 0.05 and ** *p* < 0.01 compared to control group. Data presented (**b**) are expressed as mean ± SD of experiments performed with 3 samples. +: treatment, −: no treatment.

To investigate the efficacy of Sk-EE as an anti-inflammatory agent, we used HCl/EtOH-induced gastritis mice as an in vivo model. Ranitidine, an approved drug that decreases gastric acid secretion [45], was used as the standard control compound. Under HCl/EtOH-prompted inflamed conditions, the stomachs of Sk-EE orally-injected mice presented a significant reduction in bleeding area, which is a consequence of acute inflammation, and the effect of Sk-EE was even more apparent than that of ranitidine (Figure 4a). Supported by the numerical quantification of the inflammatory lesion area (Figure 4b), Sk-EE–injected groups displayed a dose-dependent decrease in the inflamed section. To reinforce previous in vitro data, we further analyzed protein expression levels by western blotting using stomach samples from the mice of each group. Consistent with the results from Figure 3b, the expression of phosphorylated p85 and IκBα was inhibited among the Sk-EE-injected groups in a dose-dependent manner (Figure 4c).

## 4. Discussion

**Figure 5:**
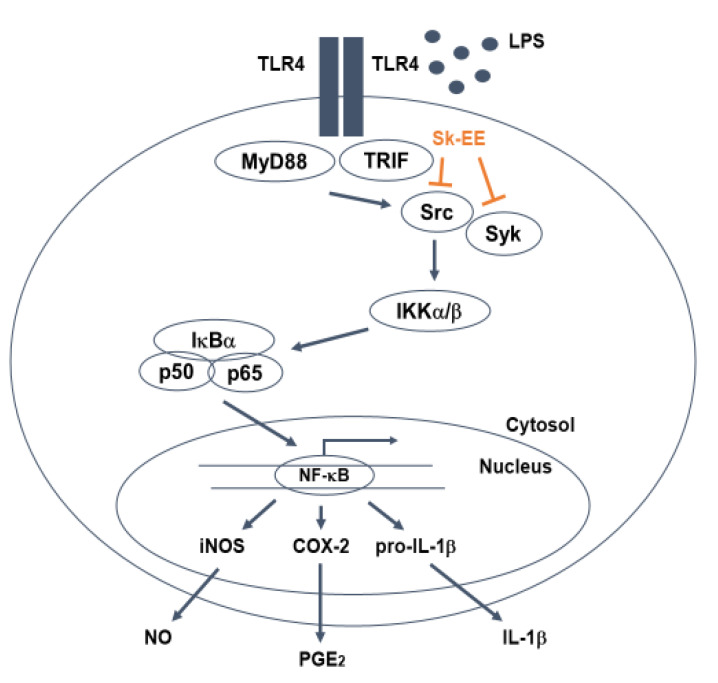
Schematic representation of overall signaling pathways and molecules targeted by Sk-EE during the inflammation process in macrophagelike cells.

In this research, we investigated the anti-inflammatory role of Sk-EE by assessing it’s in vitro and inhibitory effects in macrophages and in vivo activity using an acute gastritis mouse model. Additionally, we revealed the underlying molecular mechanisms of anti-inflammatory activity of Sk-EE (Figure 5).

NO is a reactive molecule that regulates cellular inflammatory responses through regulating various enzymes or proteins including guanylate cyclase or S-nitrosothiols [22,23,46] and is also a toxic molecule against infectious pathogens [47,48]. In our study, Sk-EE treatment significantly reduced NO production and mRNA expression of iNOS caused by Gram-negative bacteria (Figure 1a and Figure 2a). Furthermore, HPLC analysis of Sk-EE showed that it contains several types of flavonoid (Figure 1e), one of the most typical polyphenols which are secondary metabolites of plants [49]. Polyphenols, or flavonoids, were proven to have anti-inflammatory properties in that they can reduce proinflammatory cytokine production [50,51]. Supported by the inhibitory effects of detected flavonoids on NO production, we could conclude that the anti-inflammatory activity of Sk-EE is due to its phytochemical identity, which includes anti-inflammatory flavonoids.

The onset of inflammation results in the transcriptional activation of several proinflammatory cytokines and enzymes [52]. The expression of proinflammatory genes, including COX-2 and IL-1 β, was downregulated by Sk-EE (Figure 2a), implicating its anti-inflammatory effect on the transcriptional level of regulation. COX-2 is an enzyme that catalyzes the biosynthesis of PGE_2_, which is an important mediator leading to classic inflammatory signs including redness, pain, and swelling [25,53,54]. The inhibition of COX-2 by Sk-EE implies that the compound is able to prevent physiological conditions induced by inflammation. Likewise, expression patterns of PGE_2_ and observable signs of inflammation can be assessed in follow-up studies for further validation of anti-inflammatory effects. In addition, IL-1β is a proinflammatory cytokine involved in the recruitment of immune cells to the site of infection [55]. Therefore, a decrease in IL-1β by Sk-EE may prevent additional inflammatory processes induced by the recruitment of immune cells.

The expression of iNOS, COX-2, and IL-1β is commonly induced by the activation of the inflammatory transcription factor NF-κB, which regulates inflammatory responses such as cell proliferation, migration, adhesion, and lymphocyte development [13,15,20]. We validated the NF-kB activation level under inflammatory conditions; as a result, Sk-EE significantly repressed MyD88-/TRIF-induced NF-kB promoter activation (Figure 2b,c) and the nuclear translocated level of p50/ NF-κB (Figure 2d). Therefore, in parallel with previous results, we were able to conclude that Sk-EE reduced NF-κB activity and inhibited inflammation.

NF-κB signaling pathway activation is one of the most representative events following the onset of inflammation [15,20]. In NF-κB signaling, Src and Syk are the primary molecules of the NF-κB signaling pathway, which sequentially activate IKKα/β, IκBα, and NF-κB [21]. In LPS-induced macrophages, phosphorylated Syk and Src were inhibited by Sk-EE after LPS induction (Figure 3c). However, in Src- or Syk-overexpressed HEK293T cells, we observed distinct inhibitory effects of Sk-EE. The phosphorylation of Src was dose-dependently reduced by Sk-EE under Src-overexpressed conditions, while the phosphorylated Syk level remained constant (Figure 3c,d). Correspondingly, only treatment with Sk-EE of Src-overexpressed macrophages showed improved protein heat-resistance (Figure 3g). These results together imply the direct effect of Sk-EE on Src, preceding the regulation of Syk. In other words, Sk-EE directly regulates phosphorylation of Src, while it indirectly regulates Syk activation. Of note, Src has an SH2 domain, an SH3 domain, and a kinase domain [56], while Syk only has an SH2 domain and a kinase domain [57], which suggest the mechanisms of their activation or inhibition may be different from one another. For more detailed validation of the regulatory roles that Sk-EE has on Src and Syk, studies about interactions between the compound and SH domains or upstream-regulating molecules of Syk should be further performed. In addition, interaction between Syk and its possible substrate can also be investigated since Sk-EE may regulate Syk by regulating its substrate.

We also investigated the anti-inflammatory effect of Sk-EE in vitro by using a gastritis mouse model. Acute gastric inflammation commonly causes bleeding of the stomach, which appears as red lesions [58]. Therefore, we used HCl/EtOH to induce acute gastritis for in vivo assessment of the anti-inflammatory effects of Sk-EE. In acute inflammation-induced mice, inflammatory lesions of the stomach were dramatically decreased in Sk-EE–treated groups (Figure 4a). In addition, semiquantitative analysis of the lesion area showed dose-responsive reduction in Sk-EE-injected groups, even in more degrees than Ranitidine injected groups, compared to positives. Protein expression level of the gastric samples by western blotting also showed that Sk-EE treatment significantly inhibited phosphorylated, or activated, p85 and IκBα, which are two representative signaling proteins in NF-κB signaling pathway. These together suggest that the anti-inflammatory effect of Sk-EE is not limited to in vitro conditions, but is also applicable in vivo, by targeting NF-κB pathway.

Plants have been one of the most ancient remedies that people use to cure a wide range of physiological symptoms, and it is now disclosed that active ingredients of plant extracts, such as flavonoids, clearly possess anti-inflammatory or anticancer effects in mammals [59,60]. For instance, eupatilin, an active ingredient derived from *Artemisia asiatica*, was proved to have anti-inflammatory activity and approved as an anti-inflammation drug under the name of Stillen (Dong-A Pharm. Co. Korea) [61,62]. In addition, intracellular signaling molecules, including Src and Syk, have been extensively studied and are now considered as possible targets for anti-inflammatory drugs [63,64,65,66]. Likewise, the anti-inflammatory effects of Sk-EE at molecular, cellular, and physiological levels, which are hereby demonstrated, suggest the potential of this compound as a natural anti-inflammation drug with higher activity levels and less side effects relative to conventional chemicals.

## 5. Conclusions

In conclusion, we evaluated the anti-inflammatory effects of Sk-EE in vitro and in vivo by using macrophages and acute gastric-induced mice. By primarily targeting Src, Sk-EE significantly reduces protein kinases involved in the NF-κB pathway, including IKKα/β and IκBα. Consequently, nuclear translocation of p50, a subunit of NF-κB, is inhibited and results in the decrease of the transcription as well as translation of proinflammatory cytokines. All results presented in this paper together support the pharmaceutical accessibility of Sk-EE and its potential as a target of further investigation concerning the molecular regulation of various cellular responses. Finally, since exact identification of active ingredients from this extract is very important, we will employ the LC-MS method to ensure the existence of active ingredients including silibinin, genistein, quercetin, and kaempferol in the following project.

## Figures and Tables

**Table 1:** Characteristics of antibodies (host, concentration, and exposure time).

Antibody	Host	Dilution	Exposure (Time/Temperature)
p65	Rabbit	1:2500	Overnight/4 °C
p-p65	Rabbit	1:2500	Overnight/4 °C
p50	Rabbit	1:2500	Overnight/4 °C
p-p50	Rabbit	1:2500	Overnight/4 °C
Lamin A/C	Mouse	1:2500	Overnight/4 °C
p85	Rabbit	1:2500	Overnight/4 °C
IKKα	Rabbit	1:2500	2 h/RT
p-IKKα/β	Rabbit	1:2500	Overnight/4 °C
IκBα	Rabbit	1:2500	Overnight/4 °C
p-IκBα	Mouse	1:2500	2 h/RT
Syk	Rabbit	1:2500	Overnight/4 °C
p-Syk	Rabbit	1:2500	Overnight/4 °C
Src	Rabbit	1:2500	Overnight/4 °C
p-Src	Rabbit	1:2500	2 h/RT
p85	Rabbit	1:2500	2 h/RT
β-actin	Rabbit	1:2500	2 h/RT
HA	Mouse	1:2500	2 h/RT
Myc	Mouse	1:2500	2 h/RT
Rabbit IgG	Goat	1:2500	2 h/RT
Mouse IgG	Horse	1:2500	2 h/RT

RT: room temperature.

**Table 2:** Instrument and working conditions for high-performance liquid chromatography (HPLC) of quercetin, kaempherol, silibinin, and genistein.

Instrument	Condition A			Condition B		
Column	CAPCELL PAK C_18_ MG, 4.6 mm I.D. × 250 mm					
Detector	UV-Vis Detector					
Wavelength	254 nm			350 nm		
Analyzed period	30 min			40 min		
Solvent	Solvent A	2% acetic acid in water		Solvent A	0.1% formin acid in MeOH:water = 10:90	
	Solvent B	0.5% acetic acid in water:ACN = 50:50		Solvent B	0.1% formin acid in MeOH:water = 90:10	
Flow rate	1 mL/min			0.4 mL/min		
Volume	10 µL			10 µL		
Gradient	**Time (min)**	**Composition (%)**		**Time (min)**	**Composition (%)**	
		**A**	**B**		**A**	**B**
	0	28	72	0	40	60
	20	0	100	20	40	60
	30	0	100	25	70	30
	-	-	-	40	70	30

Condition A: quercetin, kaempherol. Condition B: silibinin, genistein.

**Table 3:** Sequences of polymerase chain reaction (PCR) primers used in this study.

Targets	Direction	Sequences (5′ to 3′)
*iNOS*	Forward	GGAGCCTTTAGACCTCAACAGA
	Reverse	TGAACGAGGAGGGTGGTG
*COX-2*	Forward	CACTACATCCTGACCCACTT
	Reverse	ATGCTCCTGCTTGAGTATGT
*IL-1* *β*	Forward	CAACCAACAAGTGATATTCTCCATG
	Reverse	GATCCACACACTCCAGCTGCA
*GAPDH*	Forward	CAATGAATACGGCTACAGCAAC
	Reverse	AGGGAGATGCTCAGTGTTGG
